# The antidepressant-like effect of trans-astaxanthin involves the serotonergic system

**DOI:** 10.18632/oncotarget.16069

**Published:** 2017-03-10

**Authors:** Xi Jiang, Keqi Zhu, Quanyi Xu, Guokang Wang, Jiajia Zhang, Rongrong Cao, Jiang Ye, Xuefeng Yu

**Affiliations:** ^1^ Department of Pharmacy, Zhejiang Pharmaceutical College, Zhejiang Province 315000, China; ^2^ Zhejiang University Mingzhou Hospital, Zhejiang Province 315000, China; ^3^ Ningbo First Hospital, Zhejiang Province 315000, China

**Keywords:** trans-astaxanthin, depression, serotonin (5-HT), monoamine oxidase (MAO), indoleamine 2,3-dioxygenase (IDO)

## Abstract

The antidepressant-like effect of trans-astaxanthin, a compound present rich in algae, was evaluated through behavioral and neurochemical methods. Results showed that trans-astaxanthin treatment significantly decreased the immobility time in force swim test and tail suspension test, but did not influence locomotor activity. Trans-astaxanthin treatment did not effectively antagonize hypothermia and ptosis induced by reserpine. However, pre-treatment with para-chlorophenylalanine abolished the anti-immobility effect of trans-astaxanthin in force swim and tail suspension test. These results suggested that the mechanism of antidepressant-like effect of trans-astaxanthin may involve the serotonergic system, but not noradrenaline system. This hypothesis was confirmed by neurochemical assays which showed that trans-astaxanthin increased serotonin levels in the hippocampus, frontal cortex, striatum and hypothalamus. Furthermore, our data suggested that trans-astaxanthin decreased indoleamine 2, 3-dioxygenase activity in the hippocampus, frontal cortex and hypothalamus. Inhibition of indoleamine 2,3-dioxygenase activity subsequently decreased the kynurenine/tryptophan ratio and increased the serotonin/tryptophan ratio in these brain regions. Taken together, these findings indicate that the antidepressant-like effect of trans-astaxanthin involves the serotonergic system.

## INTRODUCTION

Depression is a major cause of morbidity and mortality in children and adolescents [1]. The efficacy of monoamine modulation in treatment of depression has been widely studied [2]. Several types of classical antidepressants including selective serotonin-reuptake inhibitors (SSRIs), selective norepinephrine-reuptake inhibitors (SNRIs) and monoamine oxidase inhibitors (MAOIs) were used in clinical practice even though most of these drugs have serious adverse effects and poor therapeutic effectiveness [2]. Identification of potent and safe therapeutic agents for depression is still a significant task.

Numerous herbal medicines have been introduced into psychiatric practice because of their greater compliance and milder side effects [3]. Trans-astaxanthin, a red carotenoid pigment, is rich in algae, plants and a limited number of fungi and bacteria. It is endowed with a variety of pharmacological effects, including anti-inflammatory and antioxidant activity [4–6]. Compared with other herbal medicines, trans-astaxanthin has its advantage in locating either inside the phospholipid membrane or at the membrane surface [7] and crossing the blood-brain barrier in rodents [8]. Base on this advantage, the neuroprotective effects of trans-astaxanthin have been confirmed in several neurodegenerative disorders, such as Alzheimer’s disease and Parkinson’s disease [9–11]. More recently, our study showed that trans-astaxanthin can ameliorate lipopolysaccharide (LPS)-induced behavioral deficits, such as depressive-like behavior [12]. Moreover, previous study suggested that trans-astaxanthin can antagonize ethanol-induced depression [13]. However, the effect of trans-astaxanthin on stress-related depression and the underlying mechanism remain unclear.

The descending monoamine pathways are major targets for currently available antidepressant drugs [14]. Studies of the neurobiological mechanism underlying the interaction between stress and depression suggest the involvement of neurotransmitter systems, such as abnormalities of 5-HT, NA, DA and their metabolites [15]. 5-HT, a crucial neurotransmitter, is involved in depression pathogenesis. Abnormal 5-HT level induced by stress can cause abnormalities in behaviors such as mood, sleep, learning, memory, and sexual behavior, all of which are deranged to some extent in patients with major depression [16]. As monoamine plays a critical factor in the pathophysiology and therapy of depression, we aimed to reveal the underlying mechanism of antidepressant-like effects of trans-astaxanthin related to monoamine system in this study.

## RESULTS

### Effects of trans-astaxanthin on the immobility time of mice in the tail suspension test (TST) and the forced swim test (FST)

**Figure 1:**
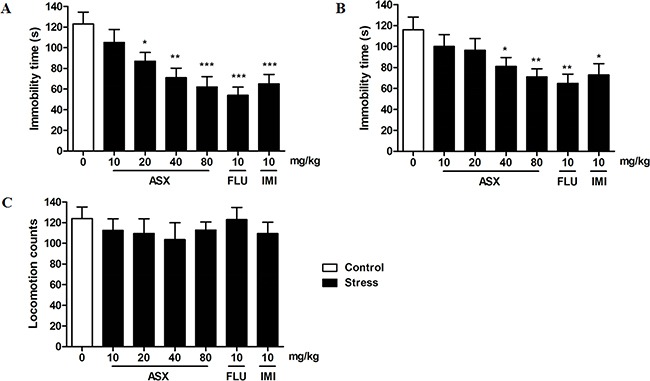
Effects of trans-astaxanthin (ASX) on the immobility time in the forced swim test (A), tail suspension test (B) and locomotor activity (C) The immobility time of force swim test and tail suspension test were assessed 1h after administration of vehicle, ASX (10, 20, 40 and 80 mg/kg, i.g.), imipramine (IMI, 10 mg/kg, i.p.) or fluoxetine (FLU, 10 mg/kg, i.p.). Values were evaluated by one-way ANOVA followed by Student-Newman-Keuls test and expressed as mean ± SEM (n=10 per group). *p<0.05, **p<0.01 and ***p<0.001 *vs*. the vehicle-treated group.

As shown in Figure 1A, a significant difference was found between the groups [F(6, 63) = 6.409, p < 0.001] in the FST. Trans-astaxanthin induced a significant decrease in immobility time in the FST. Post hoc analysis revealed that trans-astaxanthin, at doses of 20, 40 and 80 mg/kg, led to a dose-dependent reduction in the immobility period as compared to the vehicle-treated control group (p<0.05, p<0.01, p<0.001, respectively).

Similar findings were obtained in the TST [F (6, 63) = 3.334, p < 0.01]. Treatment with trans-astaxanthin (40 and 80 mg/kg, i.g.) was also shown to decrease the immobility time in the TST (p<0.05, p<0.01, Figure 1B). Furthermore, two classical antidepressants, imipramine (10 mg/kg, i.p.) and fluoxetine (10 mg/kg, i.p.), induced similar effects of decreasing the immobility time in the FST (p<0.001, p<0.001, respectively) and TST (p<0.05, p<0.01, respectively).

### Effects of trans-astaxanthin on the locomotor activity

As shown in Figure 1C, no significant difference was found among the groups in all parameters evaluated in the locomotor activity. These results indicated that trans-astaxanthin and two classical antidepressants does not decrease/increase spontaneous locomotor activity.

### Effects of descending 5-HT on the antidepressant- like effect of trans-astaxanthin in the FST and TST

**Figure 2:**
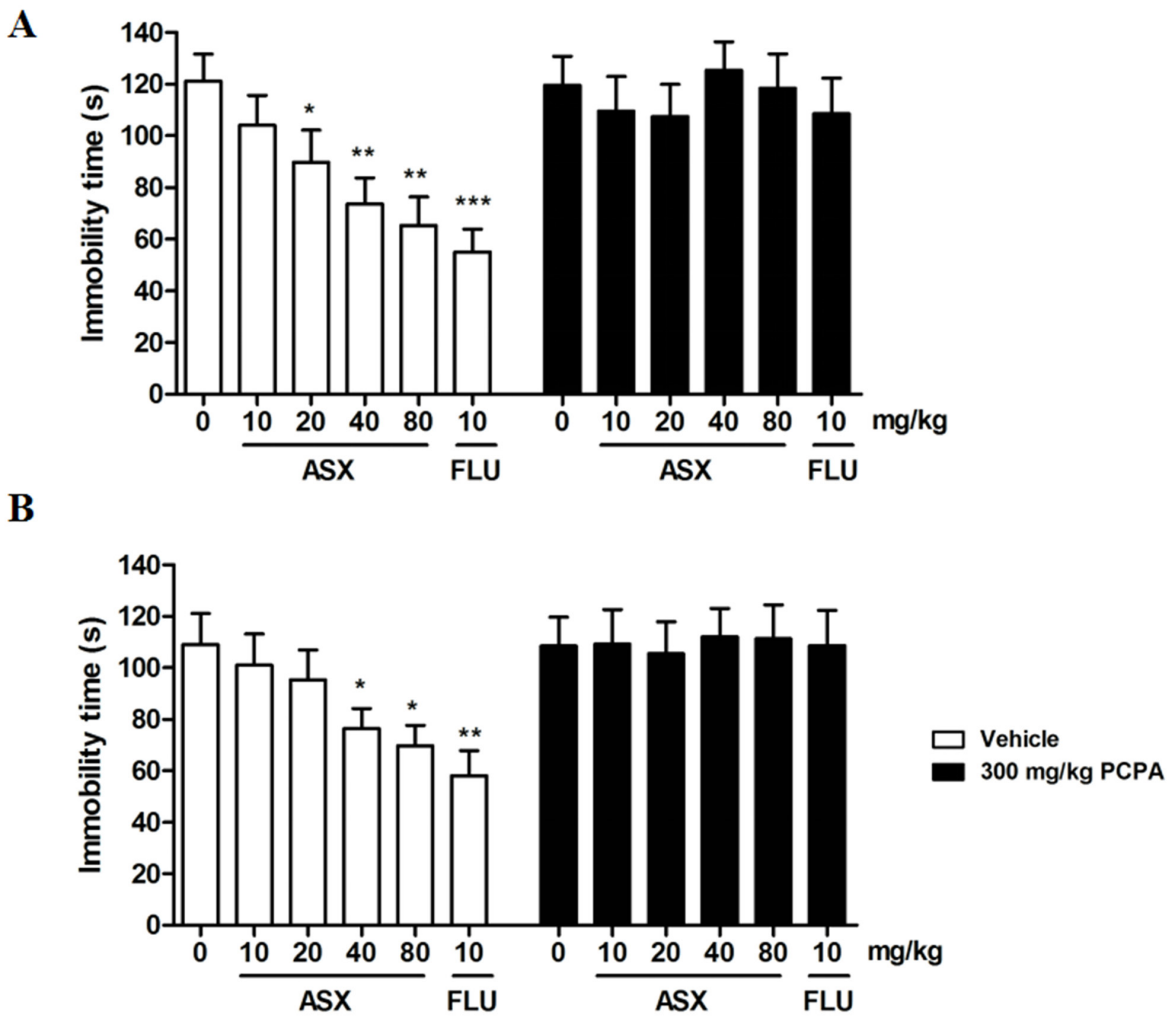
Effects of ASX on the immobility time of pre-treatment with PCPA in the forced swim test (A) and tail suspension test (B) Before ASX treatment, mice were pretreated with PCPA (300 mg/kg, i.p.) and vehicle once a day for three consecutive days. The immobility time of force swim test and tail suspension test were assessed 1h after administration of vehicle, ASX (10, 20, 40 and 80 mg/kg, i.g.), imipramine (IMI, 10 mg/kg, i.p.) or fluoxetine (FLU, 10 mg/kg, i.p.). Values were evaluated by one-way ANOVA followed by Student-Newman-Keuls test and expressed as mean ± SEM (n=10 per group). *p<0.05, **p<0.01 and ***p<0.001 vs. the vehicle-treated group.

As shown in Figure 2, trans-astaxanthin or fluoxetine significantly decreased the immobility time in the FST [F (5, 54)=5.275, p<0.001, Figure 2A] and TST [F (5, 54) =3.665, p<0.01, Figure 2B]. However, pre-treatment with PCPA (300 mg/kg, i.p.) abolished the anti-immobility effect of both trans-astaxanthin and fluoxetine in the FST [F (5, 54)=0.3047, P=0.9083] and TST [F (5, 54)=0.2996, P=0.995]. Results from these data indicated that there was an indispensable involvement of the serotonergic system in the antidepressant-like effects of trans-astaxanthin.

### Effects of trans-astaxanthin on reserpine-induced symptoms

The effects of trans-astaxanthin or imipramine on reserpine-induced hypothermia and ptosis were shown in Table 1. Reserpine at the dose of 2.5 mg/kg induced significant alteration in ptosis and hypothermia compared to the control (p < 0.001) mice group. The maximal change was found at 4 h after the injection of reserpine. However, administration of trans-astaxanthin did not prevent reserpine-induced hypothermia and ptosis.

**Table 1:** The effects of ASX on hypothermia and ptosis induced by reserpine in mice

Group	Dose (mg/kg)	Temperature (°C)		ptosis	
		0 h	4 h	2 h	4 h
Control		37.2±0.2	37.4±0.1	0	0
Reserpine	2.5	37.5±0.3	32.4±0.2***	3.7±0.3***	2.7±0.3***
ASX	10	37.5±0.1	32.7±0.1	3.6±0.2	2.6±0.2
ASX	20	37.4±0.2	33.1±0.2	3.5±0.3	2.5±0.1
ASX	40	37.1±0.3	34.5±0.2	3.1±0.2	2.1±0.3
ASX	80	37.2±0.2	34.6±0.2	2.9±0.2	2.0±0.2
IMI	10	37.4±0.2	36.9±0.3 ^###^	2.0±0.1 ^#^	1.5±0.2 ^#^

The rectal temperature and ptosis were calculated respectively after 2.5 mg/kg of reserpine administration. Values were evaluated by one-way ANOVA followed by Student-Newman-Keuls test and expressed as mean ± SEM (n=10 per group). ^***^p < 0.001 *vs*. vehicle-treated mice. ^#^ *p* < 0.05 and ^###^*p* < 0.001 *vs*. Reserpine -treated mice. ASX: trans-astaxanthin.

### Effects of trans-astaxanthin on the levels of brain monoamines and their metabolites

The levels of monoamines and their metabolites detected in the hippocampus are summarized in Table 2. 5-HT and noradrenaline levels were significantly increased after trans-astaxanthin treatment [F (6, 63) =3.21, p<0.01; F (6, 63)=2.35, p<0.05], without changing the contents of 5-HIAA, dopamine and DOPAC. The ratio of 5-HIAA/5-HT was reduced when 40 and 80 mg/kg trans-astaxanthin was administered (p<0.05, p<0.05). Moreover, 5-HT levels were increased after imipramine (10 mg/kg) or fluoxetine (10 mg/kg) treatment (p<0.01; p<0.01), but noradrenaline only increased after imipramine treatment (p<0.05).

**Table 2:** Effects of ASX on the concentrations of monoamines and their metabolites in the hippocampus of mice

Group	Dose (mg/kg)	Hippocampus (ng/g tissue)					
		5-HT	5-HIAA	5-HIAA/5-HT	Noradrenaline	Dopamine	DOPAC
Control		270.8±13.9	105.3±11.6	0.41±0.13	522.1±18.6	245.3±19.7	275.8±15.8
ASX	10	288.4±12.0	95.6±16.8	0.33±0.11	546.6±23.9	225.1±24.4	263.6±20.7
ASX	20	291.4±13.3	93.3±16.3	0.32±0.14	541.5±21.5	215.6±24.2	238.4±28.6
ASX	40	337.0±14.0*	87.6±13.7	0.25±0.09*	556.0±25.8	224.7±17.3	246.4±17.3
ASX	80	371.5±15.9**	78.4±18.3	0.21±0.1*	564.6±20.2	217.1±19.3	255.2±20.5
FLU	10	367.8±18.7**	97.5±12.4	0.26±0.17	528.7±17.6	249.4±20.7	249.3±18.7
IMI	10	364.5±19.4**	98.4±13.4	0.27±0.19	624.6±12.7**	276.4±16.8	274.6±24.5

Values were evaluated by one-way ANOVA followed by Student-Newman-Keuls test and expressed as mean ± SEM (n=10 per group). * *p* < 0.05 and ^*^ *p* < 0.01 vs. vehicle-treated mice. ASX: trans-astaxanthin.

As shown in Table 3, an increased 5-HT levels were observed in the frontal cortex after trans-astaxanthin (80 mg/kg), imipramine (10 mg/kg) or fluoxetine (10 mg/kg) treatment [F (6, 63)=5.42, p<0.01]. The noradrenaline levels were increased only after imipramine (10 mg/kg) administration. Meanwhile, the decreased ratio of 5-HIAA/5-HT was also found after higher dose of trans-astaxanthin (80 mg/kg) administration. Furthermore, no significant change in dopamine or its metabolites (DOPAC) was observed after trans-astaxanthin administration in the frontal cortex (Table 2).

**Table 3:** Effects of ASX on the concentrations of monoamines and their metabolites in the frontal cortex of mice

Group	Dose (mg/kg)	Frontal cortex (ng/g tissue)					
		5-HT	5-HIAA	5-HIAA/5-HT	Noradrenaline	Dopamine	DOPAC
Control		285.1±12.4	115.3±11.2	0.40±0.14	612.4±19.3	147.3±13.2	97.2±25.8
ASX	10	294.2±13.5	112.2±12.5	0.38±0.16	621.5±13.6	124.1±14.6	104.2±10.7
ASX	20	295.1±12.9	104.2±14.6	0.35±0.13	623.4±15.7	156.2±14.9	121.4±18.4
ASX	40	352.8±12.5*	97.5±12.8	0.27±0.15	616.2±15.4	174.7±15.3	126.2±15.3
ASX	80	394.2±16.7**	89.4±15.8	0.22±0.11*	594.6±20.2	157.1±12.3	125.4±10.7
FLU	10	384.1±15.6**	102.1±17.2	0.26±0.12	618.1±17.6	145.4±15.7	119.2±17.4
IMI	10	344.5±19.2**	96.3±15.4	0.28±0.19	754.5±16.7**	172.4±15.8	124.7±14.8

Values were evaluated by one-way ANOVA followed by Student-Newman-Keuls test and expressed as mean ± SEM (n=10 per group). * *p* < 0.05 and ^*^ *p* < 0.01 vs. vehicle-treated mice. ASX: trans-astaxanthin.

In the striatum, trans-astaxanthin, imipramine (10 mg/kg) or fluoxetine (10 mg/kg) administration induced significant increases in 5-HT levels [F (6, 63) =2.023, p < 0.05], without changing the contents of 5-HIAA, dopamine and DOPAC, and the ratio of 5-HIAA/5-HT was reduced when 80 mg/kg trans-astaxanthin was administered (p < 0.05, Table 4). Similar findings were obtained in the hypothalamus, significant increases in 5-HT levels (p < 0.05), and a decreased tendency in the ratio of 5-HIAA/5-HT was observed following trans-astaxanthin administration (80 mg/kg) (p<0.05). Imipramine and fluoxetine were also shown to increase 5-HT and/or NA levels (Table 5).

**Table 4:** Effects of ASX on the concentrations of monoamines and their metabolites in the striatum of mice

Group	Dose (mg/kg)	Striatum (ng/g tissue)					
		5-HT	5-HIAA	5-HIAA/5-HT	Noradrenaline	Dopamine	DOPAC
Control		312.3±15.1	125.5±18.4	0.41±0.15	451.5±15.2	541.2±17.3	297.5±18.9
ASX	10	324.4±17.2	122.6±15.2	0.37±0.12	431.2±17.2	541.1±14.1	261.7±17.7
ASX	20	335.7±18.9	114.5±18.4	0.34±0.15	432.1±15.9	556.5±17.8	281.5±14.9
ASX	40	361.3±18.5	104.2±18.5	0.29±0.14	462.7±18.7	564.1±18.2	259.6±25.3
ASX	80	419.5±11.7*	99.4±19.8	0.23±0.14*	482.1±19.2	537.9±16.4	268.9±16.2
FLU	10	404.2±18.6*	112.1±15.2	0.28±0.15	458.2±16.3	542.7±19.2	291.5±16.2
IMI	10	394.2±19.2*	125.1±18.4	0.31±0.14	542.2±13.7*	571.4±19.2	285.2±16.5

Values were evaluated by one-way ANOVA followed by Student-Newman-Keuls test and expressed as mean ± SEM (n=10 per group). * *p* < 0.05 vs. vehicle-treated mice. ASX: trans-astaxanthin.

**Table 5:** Effects of ASX on the concentrations of monoamines and their metabolites in the hypothalamus of mice

Group	Dose (mg/kg)	Hypothalamus (ng/g tissue)					
		5-HT	5-HIAA	5-HIAA/5-HT	Noradrenaline	Dopamine	DOPAC
Control		351.7±16.2	374.7±12.6	1.06±0.16	342.2±19.1	128.3±15.4	97.3±12.8
ASX	10	362.9±21.2	365..2±17.3	1.01±0.14	341.5±15.9	142.1±19.1	102.5±15.9
ASX	20	374.6±11.5	345.4±12.9	0.92±0.17	349.4±17.4	126.3±14.8	117.2±19.4
ASX	40	398.4±12.4	322.5±19.2	0.81±0.13	362.5±17.9	14121±18.9	109.2±16.5
ASX	80	469.2±13.7*	319.5±20.8	0.68±0.12*	381.0±12.9	134.5±16.8	98.9±19.1
FLU	10	456.3±14.6*	352.5±14.2	0.79±0.18	358.5±19.3	125.5±15.2	102.6±18.1
IMI	10	462.2±17.2*	369.3±16.4	0.82±0.16	431.1±13.7*	117.5±12.9	105.1±19.2

Values were evaluated by one-way ANOVA followed by Student-Newman-Keuls test and expressed as mean ± SEM (n=10 per group). * *p* < 0.05 vs. vehicle-treated mice. ASX: trans-astaxanthin.

### Effects of trans-astaxanthin on brain monoamine oxidase activity

Table 6 summarized the inhibition of MAO-A and MAO-B activities following treatment with trans-astaxanthin in the hippocampus, frontal cortex, striatum and hypothalamus. No significant change was found of both MAO-A and MAO-B activity after treatment with trans-astaxanthin in all four brain regions. A tendency to inhibit MAO-A activity in the hippocampus and frontal cortex can be observed only when the doses of trans-astaxanthin increased. However, the selective MAO-A inhibitor moclobemide produced monoamine oxidase-A inhibition in all four brain regions.

**Table 6:** Effects of ASX on type A and type B monoamine oxidase activities in the hippocampus, frontal cortex, striatum and hypothalamus of mice

Group	Dose (mg/kg)	Monoamine oxidase-A activity				Monoamine oxidase-B activity			
		Hippo	FC	Str	Hypo	Hippo	FC	Str	Hypo
Control		137.2±9.2	115.2±7.4	67.5±7.4	41.7±6.9	157.4±7.8	112.9±5.7	78.5±4.9	61.7±5.8
ASX	10	134.5±7.7	112.5±7.2	71.5±8.2	46.7±6.2	146.7±8.3	109.7±9.3	69.5±5.2	59.4±3.7
ASX	20	129.2±8.9	118.3±8.2	70.4±7.1	51.3±9.4	142.2±9.1	116.5±8.2	71.6±6.4	63.5±4.9
ASX	40	133.5±9.5	108.2±8.7	68.2±9.4	47.1±6.7	152.2±6.3	127.6±9.4	81.5±9.7	65.7±6.7
ASX	80	147.2±7.2	114.1±8.6	74.6±8.2	40.2±9.4	137.8±8.2	117.9±6.4	69.4±9.8	59.1±3.8
MOC	20	75.2±9.2**	67.3±7.1**	49.8±4.8*	29.8±4.9**	141.2±6.1	118.2±6.2	77.5±6.8	62.9±3.1
IMI	10	125.2±8.2	116.2±7.3	69.7±6.4	40.5±7.8	139.8±8.3	114.7±7.1	71.3±2.9	56.7±6.4
FLU	10	134.6±9.4	107.4±9.9	70.2±4.9	42.5±7.4	140.2±6.8	125.4±6.7	76.3±4.8	61.2±6.9

Values are expressed as mean ± S.E.M. with units of nmol/30 min/mg protein for 10 mice in each group. Values were evaluated by one-way ANOVA followed by Student-Newman-Keuls test. * *p* < 0.05 and ^*^ *p* < 0.01 vs. vehicle-treated mice. MOC: Moclobemide; Hippo: hippocampus; FC: frontal cortex; Str: striatum; Hypo: hypothalamus. ASX: trans-astaxanthin.

### Effects of trans-astaxanthin on brain IDO mRNA expression and ratios of brain tryptophan (TRY) metabolites

**Figure 3:**
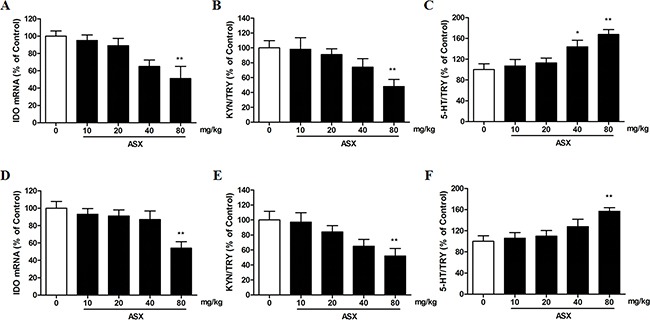
Effects of ASX on IDO mRNA expression **(A)** kynurenine (KYN)/tryptophan (TRY) ratio **(B)** and serotonin (5-HT)/TRY ratio **(C)** in the hippocamous; Effects of ASX on IDO mRNA expression **(D)** kynurenine (KYN)/tryptophan (TRY) ratio **(E)** and serotonin (5-HT)/TRY ratio **(F)** in the frontal cortex. Values were evaluated by one-way ANOVA followed by Student-Newman-Keuls test and expressed as mean ± SEM (n=10 per group). *p<0.05 and **p<0.01 vs. the vehicle-treated group.

**Figure 4:**
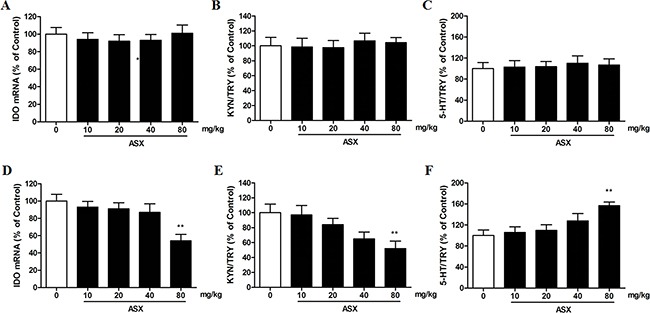
Effects of ASX on IDO mRNA expression **(A)** kynurenine (KYN)/tryptophan (TRY) ratio **(B)** and serotonin (5-HT)/TRY ratio **(C)** in the hypothalamus; Effects of ASX on IDO mRNA expression **(D)** kynurenine (KYN)/tryptophan (TRY) ratio **(E)** and serotonin (5-HT)/TRY ratio **(F)** in the striatum. Values were evaluated by one-way ANOVA followed by Student-Newman-Keuls test and expressed as mean ± SEM (n=10 per group). *p<0.05 and **p<0.01 vs. the vehicle-treated group.

As shown in Figure 3, 4, compared with saline treatment, trans-astaxanthin (80 mg/kg) specifically inhibited IDO mRNA expression in the hippocampus, frontal cortex and hypothalamus (p<0.01, Figure 3A; p<0.01, Figure 3D; p<0.05, Figure 4A).

To examine the role of IDO activity in TRY metabolism in antidepressant effect of trans-astaxanthin, we measured the levels of TRY, 5-HT, and KYN in the four brain regions using HPLC and subsequently determined the ratio of 5-HT or KYN to TRY. We observed that, compared with control group, the ratio of KYN/TRY was decreased in the hippocampus, frontal cortex and hypothalamus after trans-astaxanthin (80 mg/kg) treatment (p<0.01, Figure 3B; p<0.01, Figure 3E; p<0.05, Figure 4B), and 5-HT/TRY ratio was increased in these brain regions (p<0.01, Figure 3C for hippocampus; p<0.01, Figure 3F for frontal cortex; p<0.05, Figure 4C for hypothalamus).

Effects of trans-astaxanthin on monoamines system in four brain regions were summarized in Table 7, which suggested that trans-astaxanthin at higher dose (80 mg/kg) increased 5-HT levels in almost all tested mice brain regions. Furthermore, our data indicated that the effects of trans-astaxanthin on 5-HT levels may be due to the inhibition against IDO activity.

**Table 7:** The summarized effects of ASX on monoamines, MAO and IDO activities in brain regions of mice

Brain region	Dose (mg/kg)	5-HT	NA	DA	MAO-A	MAO-B	IDO
Hippocampus	10-80	↑	–	–	–	–	↓
Frontal cortex	10-80	↑	–	–	–	–	↓
Striatum	10-80	↑	–	–	–	–	–
Hypothalamus	10-80	↑	–	–	–	–	↓

## DISCUSSION

Knowledge of the acute mechanisms underlying the action of antidepressant drugs led to the general belief that the effective antidepressant medications act by increasing the activity of the monoaminergic system [17]. Here, we demonstrated that trans-astaxanthin was consistently effective when evaluated in two behavioral tests, and this antidepressant-like effect was accompanied by the restoration of acute stress induced alterations in brain monoamine transmitters, including serotonin and noradrenaline. Furthermore, our data also revealed that trans-astaxanthin may has an inhibitory effect on IDO activity.

The tail suspension test and forced swim test are widely used to assess depressive-like behaviors in mice because these two procedures can imitate helpless behaviors, which are frequently observed in depressed patients[18]. Our data exposed that trans-astaxanthin produces a significant inhibition of the recorded immobility time in the forced swim and tail suspension tests. We also tested the locomotor activity before FST and TST, because changes in the immobility times of FST and TST could also result from effects on locomotor activity caused by central nervous system stimulation. No significant change was found in locomotor activity test.

The descending monoamine pathways, especially noradrenergic and serotonergic transmission, are major targets for currently available antidepressant drugs [14, 19]. The present data showed that pre-treatment with PCPA prevented the anti-immobility effect of trans-astaxanthin in FST and TST. In fact, PCPA treatment at a dose of 300 mg/kg for 3 consecutive days induces a depletion of the endogenous storage of brain serotonin without influencing the noradrenaline and dopamine levels [20]. This finding indicates that serotonergic system might be involved in the antidepressant effect of trans-astaxanthin.

In parallel with the serotonergic system, the noradrenergic system is also important in the pathophysiology of depression [21]. Acute reserpine administration could induce a reversible depletion of catecholamine concentrations in noradrenergic postganglionic fibers, and leads to syndromes such as hypothermia and ptosis in mice [22]. Our data showed that trans-astaxanthin treatment could not antagonize the hypothermia and ptosis induced by reserpine.

To further disclose the mechanisms underlying the effect of trans-astaxanthin on the neurotransmitter systems, we evaluated the 5-HT, NA, DA and their metabolites levels by HPLC after trans-astaxanthin treatment. We focused on four important brain regions: hippocampus, frontal cortex, striatum and hypothalamus. They are involved in important behavioral functions, such as emotion, motivation and learning and memory [23, 24]. Our data showed that trans-astaxanthin increases the 5-HT levels in hippocampus, frontal cortex, striatum and hypothalamus, and these effects were similar to that of the positive drugs imipramine and fluoxetine. These data imply that the antidepressant-like effect of trans-astaxanthin might be due to an effect on modulation of serotonergic system, which is consistent with the behavioral changes in FST and TST when PCPA was pretreated.

Aside from 5-HT, norepinephrine is also implicated in the pathophysiology of depression [15, 21]. Norepinephrine projections innervate the limbic system, suggesting the involvement of norepinephrine in the regulation of emotions and cognition [25]. Based on the results from both the behavioral model and HPLC detection, the antidepressant-like effect of trans-astaxanthin might not be due to its influence on the regulation of noradrenergic system. However, the exact mechanism has to be further explored. In addition, dopamine concentrations were also measured and no significant change was found after trans-astaxanthin administration in the four brain regions. However, only the acute effect of trans-astaxanthin on dopaminergic systems was examined in our study, we cannot rule out the possibility that a chronic treatment with trans-astaxanthin may affect dopaminergic systems.

MAO inhibitors are known to enhance the availability of biogenic amines (5-HT, NA or DA) at the synapse [26–28]. MAO-A is involved in metabolism of 5-HT and NA, while MAO-B preferentially metabolizes the dopamine neurotransmitter[29]. Our data showed that neither MAO-A nor MAO-B activity was decreased after trans-astaxanthin treatment. These results indicated that the monoamine neurotransmitter levels increased after trans-astaxanthin administration might not be due to the inhibition of monoamine hydrolysis. In fact, not only the hydrolysis process, but also the synthesis of monoamine neurotransmitter plays an important role in depression. 5-HT is synthesized via tryptophan (TRY) pathway. Recently, a low TRY level was observed in depressed animal model [30]. Furthermore, indoleamine 2, 3-dioxygenase (IDO) activation was found in the development of depressive-like behavior [31, 32]. IDO is a rate-limiting enzyme in TRY metabolism. IDO activity is associated with the contents of 5-HT, kynurenine (KYN), and neuroplastic changes, which are all associated with depression [33, 34]. The importance of the activation of IDO in the pathophysiology of depression is also supported by the evidence that the blockade of IDO inhibits the onset of the lipopolysaccharide-induced depressive-like behavior in mice [31]. In order to verify whether the increase in 5-HT levels after trans-astaxanthin treatment is resulted from the inhibition of IDO activity, we firstly detected IDO mRNA expression in the four brain regions. Results exhibited that IDO mRNA expression were inhibited in all tested brain regions after trans-astaxanthin administration. Moreover, our study found decreased KYN/TRY ratio and increased 5-HT/TRY ratio in these brain regions after trans-astaxanthin treatment. Indeed, inadequate endogenous 5-HT levels and detrimental increases in the concentration of KYN derivatives lead to depressive symptoms [30, 35, 36]. Thus, the antidepressant-like effects of trans-astaxanthin in mice may be associated with its regulation of IDO activation. Accurately, trans-astaxanthin normalize the increased KYN/TRY ratio and decreased 5-HT/TRY ratio caused by IDO activity in the brain.

In summary, the current study describes how trans-astaxanthin exerts antidepressant-like effects in behavioral despair tests. This positive effect is likely mediated via the central serotonergic system by inhibiting the IDO activity, and is also related to noradrenergic system. Further studies will focus on the change of receptors and signal pathways after chronic treatment with trans-astaxanthin to identify its pharmacotherapeutic potentials.

## MATERIALS AND METHODS

### Animals

Male ICR mice (4-6 weeks, 20–22 g) were obtained from the Animal Center of Shanghai Branch, Chinese Academy of Sciences. Upon arrival, the mice were housed eight per cage and acclimatized to a colony room with controlled ambient temperature (22±1 °C), humidity (50±10%) and a 12 hour light/dark cycle. A standard diet was fed and water was provided ad libitum. They acclimated 7 days before entry into the subsequent study. The experiments were performed with 10 mice per treatment group according to a randomized schedule. All experiments were conducted in accordance with the National Institutes of Health Guide for Care and Use of Laboratory Animals, and approved by Wenzhou Medical University Committee on Animal Care and Use.

### Drug and drug administration

Trans-astaxanthin, imipramine hydrochloride, p-chlorophenylalanine HCl (PCPA, an inhibitor of serotonin synthesis), apomorphine hydrochloride, kynuramine dihydrobromide, 4-hydroxyquinoline, clorgyline, deprenyl, 5-hydroxytryptamine, noradrenaline, dopamine, 5-hydroxyindoleacetic acid (5-HIAA) and 4-dihydroxyphenylacetic acid (DOPAC) were purchased from Sigma Chemical Co. (USA). Moclobemide hydrochloride and sodium carboxymethyl cellulose were provided by Beijing Institute of Pharmacology and Toxicology (China). For oral administration (via gavage, i.g.), trans-astaxanthin was dissolved in 0.5% sodium carboxymethyl cellulose and moclobemide was dissolved in redistilled water. For intraperitoneal injection, imipramine and fluoxetine were dissolved in redistilled water. In acute experiments, the behavioral and neurochemical tests were conducted 1 h after trans-astaxanthin (20, 40, 80 mg/kg, i.g.) treatment [37]. The effects of positive antidepressants such as moclobemide (20 mg/kg, i.g.), imipramine (10 mg/kg, i.p.) and fluoxetine (10 mg/kg, i.p.) were tested 1 h (meclobemide) and 30 min (imipramine and fluoxetine) respectively, after administration of the drugs as previously described [20, 38].

### Force swim test

Mice were submitted to FST after trans-astaxanthin treatment (n=10/group, different from those used for TST). The forced swim test employed was similar to that described previously [18, 23, 39]. Briefly, the mouse had a swimming-stress session for 15 min (pre-test) in the glass cylinder (height: 25 cm; diameter: 10 cm; containing 10 cm of water at 24 ± 1 °C). One hour later, the mouse was placed in the cylinder again for a period of 6 min (test session), the last 4 min of the 6-min test was scored for the immobility time (floating with only small movement necessary to keep the head above water).

### Tail suspension test

Mice were submitted to TST after trans-astaxanthin treatment (n=10/group, different from those used for FST). The tail suspension test was carried out as previously described [40] with minor modification [38]. In brief, the mouse was suspended 50 cm above the floor using adhesive tape, placed approximately 1 cm from the tip of the tail. Each mouse is tested only once and black baffle plate was used to avoid of view from the other mice. Then immobility time was recorded during a test period of 6 min. Mice were considered immobile only when they hung passively and completely motionless.

### Locomotor activity

The assessment of locomotor activity was carried out as previously described [40]. Briefly, locomotor activity of mouse was measured by an experimental instrument with five activity chambers (JZZ98, Institute of Materia Medica, Chinese Academy of Medical Sciences, China). Mice were placed in the chambers and their paws contacted or disconnected the active bars producing random configurations that were converted into pulses. The pulses, which were proportional to the locomotor activity of the mice, were automatically recorded as the cumulative total counts of motor activity. Mice performed a training session for 5 min (pre-test), and then locomotion counts were recorded during the 10-min testing period. Each mouse received locomotor activity test before FST or TST.

### Depletion of descending serotonin (5-HT)

To study the role of serotonergic system in the antidepressant like effect of trans-astaxanthin, the mice were injected intrathecally with PCPA for denervation of 5-HT before trans-astaxanthin treatment. PCPA was dissolved in 0.9% physiological saline. For 5-HT depletion, PCPA at a dose of 300 mg/kg was treated (i.p.) once a day for three consecutive days [18, 41]. On the fourth day, the forced swim and tail suspension tests were performed 30 min after trans-astaxanthin administration.

### Reserpine test

The reserpine test was performed according to the method described by Bourin et al [42]. In brief, the animals were injected intraperitoneally with 2.5 mg/kg of reserpine. Hypothermia was estimated at two time points, 0 h (before any drug administration) and 4 h after reserpine injection. The ambient room temperature was maintained at 22±1 °C in the duration of the experiment. The degree of ptosis in each mouse was recorded at 2 h and 4 h after reserpine injection. The following rating scale was used to assess the degree of ptosis: 0, eyes open; 1, eyes one-quarter closed; 2, eyes half closed; 3, eyes three-quarters closed; 4, eyes completely closed.

### HPLC

Mice were decapitated and their brains were rapidly removed and frozen on dry ice. Various brain areas, including the frontal cortex, hippocampus, hypothalamus and striatum, were dissected on a cold plate [43]. The tissue samples were weighed and stored at −80 °C until homogenization.

The contents of 5-HT, noradrenaline, dopamine, 5-HIAA, DOPAC tryptophan and kynurenine were measured as described previously [44] by high-performance liquid chromatography (HPLC) with electrochemical detection. Each frozen tissue sample was homogenized by ultrasonication in 200 μl of 0.4 M perchloric acid (solution A). The homogenate was kept on ice for 1 h and then centrifuged at 12,000 g and 4 °C for 20 min. The pellet was discarded. An aliquot of 160 μl of supernatant was added to 80 μl of solution B (0.2 M potassium citrate, 0.3 M dipotassium hydrogen phosphate and 0.2 M EDTA). The mixture was kept on ice for 1 h and then centrifuged at 12,000 g and 4 °C for 20 min again. 20 μl of the resultant supernatant was directly injected into an ESA liquid chromatography system equipped with a reversed-phase C18 column (150×4.6 mm I.D., 5 μm) and an electrochemical detector (ESA CoulArray, Chelmstord, MA, USA.). The mobile phase (pH = 3.0) consisted of 75 mM NaH_2_PO4, 25 μM EDTA (disodium salt), 1.7 mM octanesulfonic acid, and 100μl/L triethylamine in acetonitrile:water (7:93 v:v). The flow rate is 1 ml/min. The protein concentrations were calculated by the Bradford assay.

### MAO assay

The MAO activity was analyzed according to our established protocol with minor modifications [45]. The brain tissues were homogenized with 4 ml of phosphate buffer (pH=7.4). The activities of monoamine oxidase-A and -B in brain tissues were measured in the presence of 1 mM clorgyline (type A inhibitor) or deprenyl (type B inhibitor). To lyse the membranes, the tissue homogenate was treated with 0.4 ml of 20% Triton X-100. Subsequently, 2.5 ml of phosphate buffer (pH 7.4) was mixed with 0.2 ml of the tissue homogenate. The mixture was then incubated at 37 °C for 15 min. Then 30 μl of 2.19 mM kynuramine dihydrobromide was added to the reaction mixture as substrate. The reaction was kept at 37 °C for 30 min and stopped by adding 0.2 ml of 5 M perchloric acid. After centrifuging at 1500 g for 15 min, an aliquot of 0.5 ml of the supernatant was added to 2.5 ml of 1 M NaOH. The fluorescent intensity was detected with excitation at 315 nm and emission at 380 nm using a fluorescent spectrometer. The concentration of 4-hydroxyquinoline was calculated based on the standard curve and the MAO activity was expressed as nmol of 4-hydroxyquinoline formed/30 min/mg protein.

### Quantitative real-time RT-PCR

Total cellular RNA was isolated using Trizol reagent (Trizol Invitrogen) according to the manufacturer’s protocol and RNA (1 mg) was reverse transcribed using MJ MiniTM Gradient Thermal Cycler (Bio-Rad, Hercules CA, USA). RNA concentration was determined using a spectrophotometer (Bio-Rad. Labs) at 260 nm. The PCR reaction was performed using iCycler Real-Time PCR machine (Bio-Rad, Hercules CA, USA). SYBR Green (iQ SYBR Green supermix reagent, Bio-Rad) was added to each sample at a concentration of 50 nmol/L. Protocol of the real-time PCR was as follows: initial denaturation at 95 °C for 10 min, followed by 40 cycles at 95 °C for 10 s, 58 °C for 30 s. At the end of the PCR reaction, a melting curve was obtained by holding at 95 °C for 15 s, cooling to 60 °C for 1 min, and then heating slowly at 0.5 °C /s until 95 °C. The primers: IDO: Forward (5′-AGC ACT GGA GAA GGC ACT GT-3′); Reverse (5′-ACG TGG AAA AAG GTG TCT GG-3′); β-actin: Forward (5′-TGG AAT CCT GTG GCATCC ATG AAA C-3′); Reverse (5′-AA AAC GCA GCT CAG TAA CAGTCC G-3′). PCR products were amplified in the real-time PCR machine followed by melt curve analysis and gel electrophoresis to verify specificity and purity of product. All the data were normalized to the housekeeping gene, β-actin.

### Statistical analysis

Results were presented as the mean ± S.E.M. All data were carried out by one-way analysis of variance (ANOVA), followed by post hoc Student-Newman-Keuls test. Differences were considered significant if p < 0.05.

## Abbreviations

SSRIs: Selective serotonin-reuptake inhibitors
SNRIs: selective norepinephrine-reuptake inhibitors
MAOIs: monoamine oxidase inhibitors
LPS: lipopolysaccharide
TST: tail suspension test
FST: forced swim test
DOPAC: dopamine or its metabolites
TRY: tryptophan
KYN: kynurenine
5-HIAA: 5-hydroxyindoleacetic acid
DOPAC: 4-dihydroxyphenylacetic acid
